# Effect of vitamin D supplementation on blood lipids in patients with metabolic syndrome: a meta-analysis

**DOI:** 10.7717/peerj.21086

**Published:** 2026-04-13

**Authors:** Qijun Wang, Hong Huang, Wenshu Jin, Shaoguan Wang, Xiaofang Tu

**Affiliations:** Department of Endocrinology, Zhejiang Hospital, Hangzhou City, Zhejiang Province, China

**Keywords:** Vitamin D, Lipids, Metabolic syndrome, Triglyceride, Cholesterol

## Abstract

**Background:**

Vitamin D, a fat-soluble vitamin critical for bone metabolism, has recently been implicated in metabolic homeostasis. Growing evidence suggests its potential role in modulating lipid profiles in metabolic syndrome.

**Objectives:**

This meta-analysis evaluated the effects of vitamin D supplementation on blood lipids in patients with metabolic syndrome.

**Methods:**

We searched the randomized controlled trials from 1948 to February 1, 2026 involving vitamin D supplementation treatment for patients with metabolic syndrome from PubMed, EMBASE, Cochrane library, SinoMed, and conducted a meta-analysis. The meta-analysis was registered on the PROSPERO, the registration number is CRD42024591657.

**Results:**

Finally, seven trials were included in the meta-analysis. The results demonstrated that vitamin D supplementation significantly reduced triglyceride levels (MD = −12.64, 95% CI [−21.23∼−4.04], *P* = 0.004). However, no significant differences were observed in total cholesterol or low-density lipoprotein cholesterol levels between the vitamin D treated groups and control groups. Notably, vitamin D supplementation showed potential benefits in increasing high-density lipoprotein cholesterol levels particularly with daily regimens (MD = 1.77, 95% CI [0.90∼2.64] *P* < 0.0001, I^2^ = 35%).

**Conclusions:**

Our meta-analysis confirmed the effects of vitamin D in the management of triglyceride level in metabolic syndrome patients. Vitamin D supplementation showed potential benefits on increasing the high-density lipoprotein cholesterol levels especially with daily regimens. More randomized controlled trials are needed to confirm the result.

## Introduction

Vitamin D, a fat-soluble prohormone, is either endogenously synthesized from 7-dehydrocholesterol (7-DHC) in the skin upon ultraviolet B radiation exposure, or obtained through dietary sources and supplements (Benedik, 2021). To become biologically active, vitamin D undergoes sequential hydroxylation in the liver and kidney. In modern societies, widespread vitamin D deficiency has emerged as a critical global health concern (Płudowski et al., 2023), attributable to reduced outdoor activities and application of extensive skin coverage. Notably, this deficiency persists across all age groups and geographical regions, including low-latitude areas and industrialized nations with established vitamin D fortification programs (Palacios & Gonzalez, 2014), underscoring the growing importance of supplementation strategies.

Traditionally recognized for its pivotal role in bone homeostasis through calcium and phosphorus metabolism regulation, vitamin D supplementation has become a cornerstone in osteoporosis management. However, emerging evidence has revealed its pleiotropic effects extending far beyond skeletal health. Vitamin D is associated with cardiovascular disease (Rendina et al., 2022), and energy metabolism. Vitamin D deficiency, for example, is associated with diabetes (Zaromytidou et al., 2022), atherosclerosis (Bener et al., 2021), and high blood pressure (Sheikh et al., 2020). Importantly, intervention studies suggest that vitamin D supplementation may confer therapeutic benefits for glycemic control (Mehta et al., 2022), blood pressure regulation (Sheikh et al., 2020), and body weight control (Xenos et al., 2022) which are core components of metabolic syndrome (MetS). Therefore, investigating vitamin D supplementation strategies in individuals with metabolic syndrome is warranted.

Metabolic syndrome is a cluster of several known cardiovascular risk factors, including obesity, hypertension, and disorders of glucose and lipid levels. MetS is highly prevalent in many areas (Beckett et al., 2023). While numerous studies have investigated vitamin D’s effects on glucose metabolism, its impact on lipid profile—a core component of MetS—remains contentious (Alves et al., 2021; Ebadi et al., 2021; Rajaie et al., 2021). To address this knowledge gap, we conducted a systematic meta-analysis of randomized controlled trials to clarify this controversy and quantify the effect of vitamin D supplementation on lipid profiles among patients with MetS. Among the blood lipid parameters, both triglyceride (TG) and high-density lipoprotein cholesterol (HDL-C) are core indicators of metabolic syndrome. Given the potential acute risks of elevated TG (*e.g.*, acute pancreatitis) and its high prevalence in the population with metabolic syndrome, TG was selected as the primary outcome in this meta-analysis.

## Materials and Methods

### Data source and search strategy

A comprehensive search strategy was employed to identify randomized controlled trials (RCTs) investigating the effects of vitamin D supplementation on blood lipids in individuals with metabolic syndrome, across the EMBASE, PubMed, Cochrane, and SinoMed platforms. The electronic search covered the period from January 1, 1948 to February 1, 2026. The literature search was conducted in two phases: an initial search (January 1, 1948 to November 9, 2024) and an update search (November 9, 2024 to February 1, 2026) performed at the editor’s request to incorporate the most recent evidence. The search strategy was developed in accordance with the Cochrane Handbook for Systematic Reviews of Interventions and relevant published studies. The detailed search strategy was affixed (Strategy S1).

### Study selection criteria

Studies were selected based on the following predefined PICOS criteria: (1) Population: patients diagnosed with metabolic syndrome; (2) Intervention: vitamin D supplementation (any form, dose, or duration); (3) Comparison: placebo or no vitamin D supplementation, with control groups receiving all interventions identical to the experimental group except for vitamin D. (4) Outcome: primary outcome—TG levels; Secondary outcomes—total cholesterol (TC), HDL-C, and low-density lipoprotein cholesterol (LDL-C); (5) study design: randomized controlled clinical trials. Qijun Wang and Xiaofang Tu assessed the eligibility of the studies independently, with discrepancies resolved through discussion or consultation with a third reviewer Wenshu Jin.

### Data extraction

Two researchers independently extracted the data of study characteristics, participant demographics (including body mass index, age, gender, *etc.*), intervention details, lipid levels with baseline and post-intervention values, using a standardized form. The details of the data are shown in Tables 1 and 2. The recorded outcomes contained the TG, TC, HDL-C and LDL-C. Disagreement was resolved by discussing with the third author.

### Quality assessment

The quality of the included studies was mainly evaluated by two independent authors based on the following seven aspects: (1) allocation generation; (2) allocation concealment; (3) blinding of participants, investigators, and examiners; (4) the number of patients lost to follow-up; (5) intention-to-treat (ITT) analysis; (6) selective reporting; (7) other factors that could introduce bias, such as the baseline comparability of groups.

### Statistical analysis

The meta-analysis was conducted using the software Review Manager 5.4. Risk ratio was calculated for dichotomous variables and mean difference (MD) for the continuous variables. Post-intervention values were extracted for meta-analysis. But when baseline imbalances existed in a study, change-from-baseline data were used to correct bias. If the mean value was unavailable, it was estimated along with the standard deviation (SD) using the median and range, following the method described by Hozo et al. (2005). Treatment groups receiving different doses of vitamin D within the same study were pooled for analysis. However, considering the potential interaction of interventions, groups were not combined in studies employing a factorial design. For each analysis, heterogeneity was estimated using the *χ*^2^ test and I^2^ metrics. Significant heterogeneity was indicated by *P* < 0.1 or I^2^ > 50%, in which case reasons for obvious heterogeneity were investigated, and a random effects model was chosen to analyze the combined results, otherwise a fixed effects model was employed.

**Table 1:** The characteristics of included studies.

**Study**	**BMI** **(kg/m** ^**2**^ **)**	**Location**	**Length** **(weeks)**	**Age** **(years)**	**Sex** **(M/F)**	**Participants**	**Treatment group**		**Control group**	
							**Intervention**	***N***	**Intervention**	***N***
Farag et al. (2019)	32.96 ± 5.11	Iraq	12	41.59	21/28	MetS 30–50 years old	2,000 IU/d VitD	24	Only placebo	25
Kelishadi et al. (2014)	27.94 ± 1.05	Isfahan	12	10–16 years	–	MetS 10–16 years old	Total 300,000 IU VitD +recommendations	21	Placebo + recommendations	22
Makariou et al. (2017)	32.2 ± 5.60	Greece	12	51.5	26/24	MetS	2,000 IU/d VitD+ dietary instructions	25	Only dietary instructions	25
Nazarabadi et al. (2022)	34.62 ± 1.11	Kermanshah	8	55.78	0/23	MetS, postmenopausal women 10–20 ng/mL 25(OH)D	50,000 IU/wk. VitD	12	Placebo	11
Salekzamani et al. (2016)	33.38 ± 4.56	Tabriz	16	40.49	35/36	MetS	50,000 IU/wk. VitD	35	Placebo	36
Wongwiwatthananukit et al. (2013)	26.80 ± 3.79	Thailand	8	63.65	46/44	MetS >20 years old VitD deficiency	20,000–40,000 IU/wk. VitD_2_	60	Placebo	30
Yin et al. (2016)	27.1 ± 3.4	Jinan	48	49.5	68/58	MetS and VitD deficiency 35–60 years old TG≤5.5 mmol/l	600 mg/d Ca+ 700 IU/d VitD	61	600 mg/d Ca+ Placebo	62

**Notes.**

BMI body mass index. BMI was expressed as mean ± SD Length the duration of follow-up N sample size MetS metabolic syndrome VitD vitamin D

**Table 2:** Methodological quality of included studies.

**Study**	**Allocation generation;** **concealment**	**Blinding**			**Follow-up lost (n)**	**ITT**	**Selective reporting**	**Unequal baseline or other remarks**
		**Participants**	**Investigators**	**Examiners**				
Farag et al. (2019)	B, B	NA	NA	NA	11	N	N	TG; HDL-C
Kelishadi et al. (2014)	A, B	Y	Y	Y	7	N	N	N
Makariou et al. (2017)	A, B	N	N	NA	0	Y	N	N
Nazarabadi et al. (2022)	A, B	Y	N	NA	2	N	NA	N
Salekzamani et al. (2016)	A, A	Y	Y	Y	9	N	N	TG
Wongwiwatthananukit et al. (2013)	B, B	Y	Y	NA	6	Y	N	N
Yin et al. (2016)	B, B	NA	NA	NA	3	N	N	N

**Notes.**

ITT intention to treat analysis A adequate B unknown N no Y yes NA unable to assess TG triglyceride HDL-C high-density lipoprotein cholesterol

## Results

**Figure 1:**
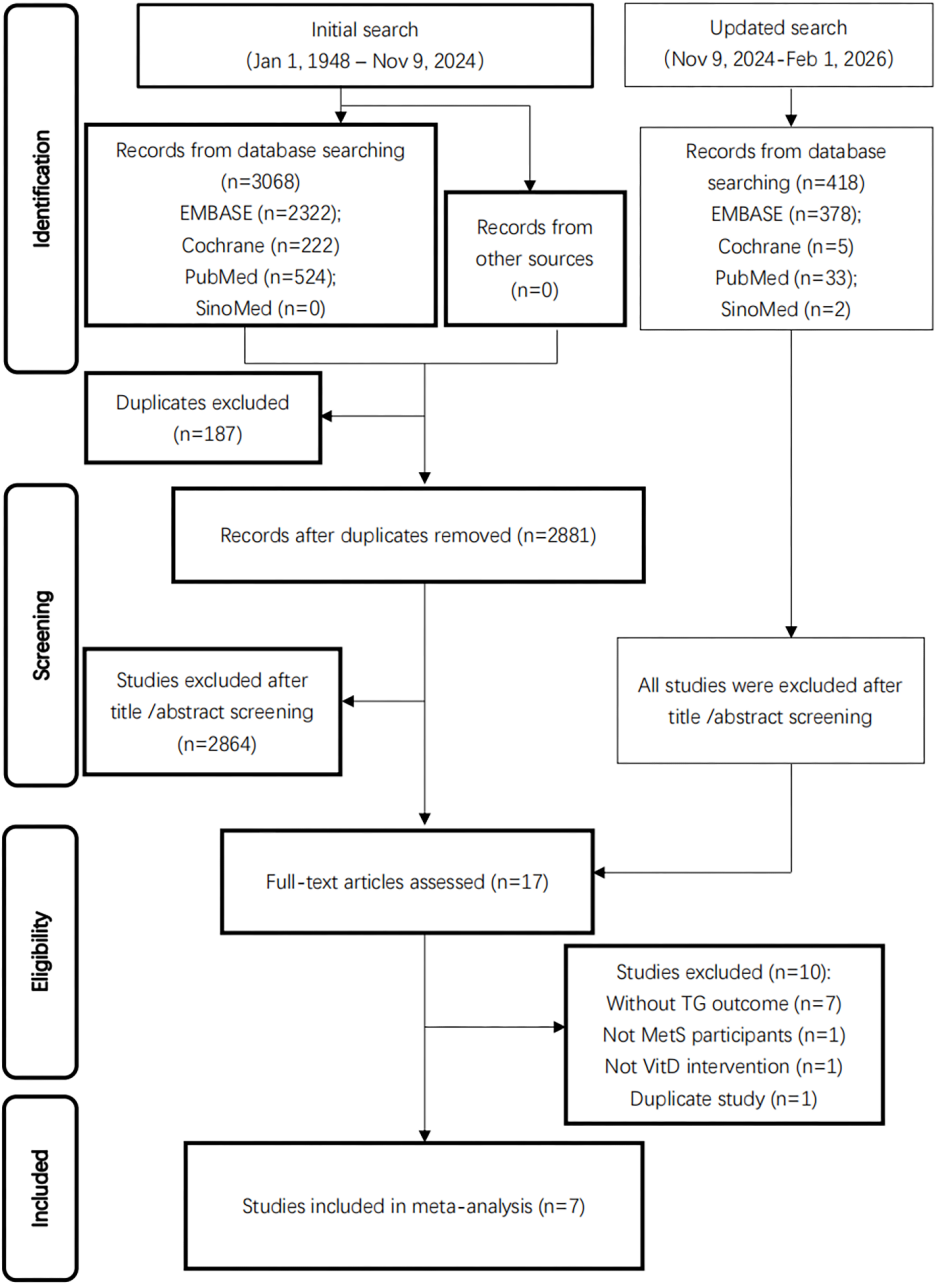
PRISMA diagram of study selection.

A total of 3,486 studies were identified using the predefined research strategy (Strategy S1). And seven trials ultimately met the inclusion criteria (Farag et al., 2019; Kelishadi et al., 2014; Makariou et al., 2017; Nazarabadi et al., 2022; Salekzamani et al., 2016; Wongwiwatthananukit et al., 2013; Yin et al., 2016). The PRISMA flow diagram (Fig. 1) details the screening process. No additional studies meeting inclusion criteria were identified in the update search.

### The characteristics and quality assessment

The characteristics of included studies were shown in the Table 1. All seven included studies enrolled patients with MetS. Although the definition of MetS varied slightly across international standards, it was generally similar and based on the following five aspects: waist circumference, TG, blood glucose, blood pressure, and HDL-C levels. The cut-off point values for waist circumference varied slightly among different standards. As there is no universal definition of MetS in the pediatric age group, a continuous MetS score, also based on the five aspects above, was used in study (Kelishadi et al., 2014).

The mean age of patients in the studies ranged from 40.49–63.65 years, with the exception of the Kelishadi et al. (2014) study, which included younger participants aged 10–16 years. In addition, the study (Nazarabadi et al., 2022) only included postmenopausal women participants.

The methodological quality of the included studies is summarized in Table 2. All of the studies reported randomization, but two studies exhibited baseline imbalances between the intervention and control groups.

### Different regimens of vitamin D treatment and vitamin D improvement

Vitamin D regimens varied across the included trials, as presented in Tables 1 and 3. Most studies had durations of 8–16 weeks, except for Yin et al. (2016) that extended to 48 weeks. Dosages of vitamin D ranged from 700 IU per day to 50,000 IU per week. The mean or median values of baseline 25-hydroxyvitamin D (25(OH)D) levels of included participants were all below 20 ng/ml. Across different vitamin D treatment regimens, the reported 25(OH)D levels in the treatment groups consistently increased to over 20 ng/mL, with five trials even achieving levels above 30 ng/mL.

### Effect of vitamin D supplementation on TG level

**Figure 2:**
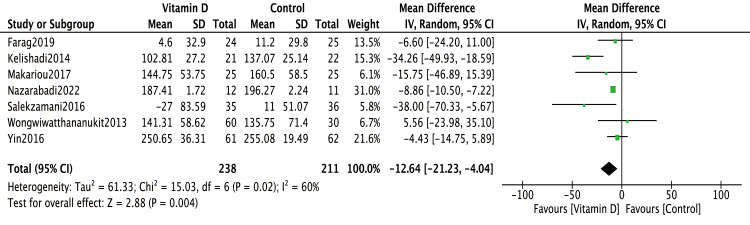
Meta-analysis of effects of vitamin D on TG. The baseline TG levels were significantly different between intervention and control groups in Farag et al. (2019) and Salekzamani et al. (2016) thus, the change-from-baseline values were used to correct the bias. TG, triglyceride; SD, standard deviation.

All seven studies compared TG levels in metabolic syndrome patients with and without vitamin D supplementation. The meta-analysis revealed that vitamin D supplementation significantly reduced TG levels (MD = −12.64, 95% CI [−21.23∼−4.04], *P* = 0.004) (Fig. 2). However, significant heterogeneity was observed (I^2^ = 60%). Exclusion of the Kelishadi et al. (2014) study eliminated heterogeneity (I^2^ = 0%) and resulted in an even more pronounced reduction in TG levels (MD = −8.78, 95% CI [−10.39∼−7.17], *P* < 0.00001).

**Figure 3:**
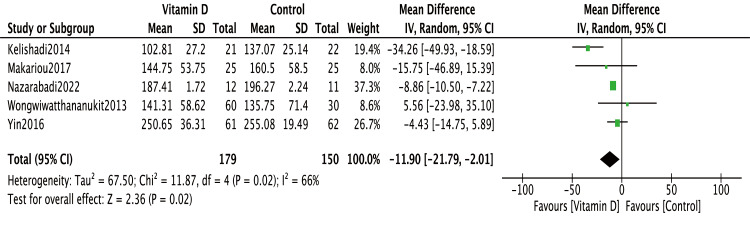
Subgroup analysis of effects of vitamin D on TG. Farag et al. (2019) and Salekzamani et al. (2016) were excluded because of the unbalanced TG levels between the intervention and control groups at baseline. TG, triglyceride; SD, standard deviation.

In Farag et al. (2019) and Salekzamani et al. (2016), baseline TG levels were significantly higher in the treatment groups than in the control groups. Therefore, change-from-baseline values were used for meta-analysis in these two studies. Notably, even after excluding these two studies, the overall conclusion remained unchanged (MD = −11.9, 95% CI [−21.79∼−2.01], *P* = 0.02) (Fig. 3).

**Table 3:** Participants’ vitamin D levels before and after interventions.

**Study**	**25(OH)D (ng/ml)** ^a^				**Vitamin D dose in treatment groups**
	**Treatment group**		**Control group**		
	**Baseline**	**After**	**Baseline**	**After**	
Farag et al. (2019)	10.75 ± 2.79	23.2 ± 4.9	12.16 ± 3.95	12.6 ± 4	2,000 IU/d
Kelishadi et al. (2014)	18.27 ± 2.04	32.01 ± 2.14	17.91 ± 2.27	19.07 ± 2.01	Total 300,000 IU
Makariou et al. (2017)	16 (3–35)^b^	30.6 (8.4–67.0)^b^	10 (4–39.6)^b^	13.0 (3.5–37.0)^b^	2,000 IU/d
Nazarabadi et al. (2022)	–	–	–	–	50,000 IU/wk
Salekzamani et al. (2016)	6.58 ± 6.20^c^	31.35 ± 8.68^c^	9.39 ± 8.54^c^	8.58 ± 7.10^c^	50,000 IU/wk
Wongwiwatthananukit et al. (2013)	15.08 ± 3.16	26.8 ± 6.37	16.20 ± 2.99	18.99 ± 6.71	20,000 IU/wk
	14.29 ± 3.35	30.03 ± 6.97			40,000 IU/wk
Yin et al. (2016)	14.6 ± 2.18	33.1 ± 4.37	14.2 ± 2.55	14.6 ± 2.8	700 IU/d

**Notes.**

a Date are presented as mean ± SD if not otherwise stated.

b Data are presented as median (range, min-max).

c The original value was divided by 2.5 to convert the value of 25(OH)D from nmol/l to ng/ml. 25(OH)D: 25-hydroxyvitamin D.

The number of studies included in this meta-analysis was relatively small, thus making the assessment of publication bias difficult. Nevertheless, we provided a visualized funnel plot for reference only (Fig. S1).

### Effect of vitamin D supplementation on cholesterol

No significant differences were observed in TC (MD = −6.04, 95% CI [−16.83∼4.74], *P* = 0.27) (Fig. S2), HDL-C (MD = 1.41, 95% CI [−1.51∼4.33], *P* = 0.34) (Fig. S3), or LDL-C (MD = −4.43, 95% CI [−11.11∼2.25], *P* = 0.19) (Fig. S4) levels between groups with and without vitamin D supplementation.

**Figure 4:**
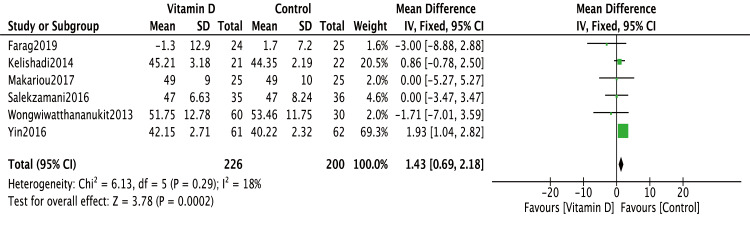
Meta-analysis of effects of vitamin D on HDL-C level. Nazarabadi et al. (2022) was excluded from meta-analysis. The baseline HDL-C levels were significantly different between intervention and control groups in Farag et al. (2019) thus, the change-from-baseline values were used in this study to correct the bias. HDL-C, high-density lipoprotein cholesterol; SD, standard deviation.

**Figure 5:**
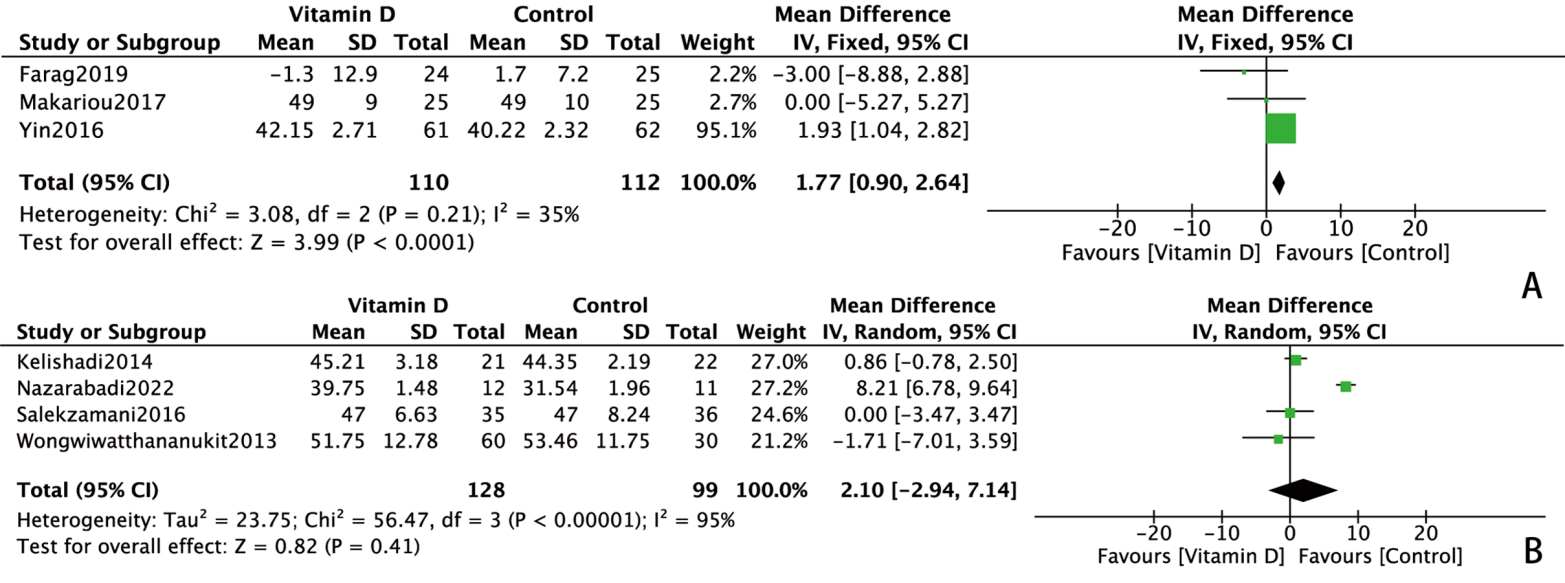
Subgroup analysis of effects of vitamin D on HDL-C. (A) Daily regimens of vitamin D supplementation. (B) Weekly regimens of vitamin D supplementation. The baseline HDL-C levels were significantly different between intervention and control groups in Farag et al. (2019) thus, the change-from-baseline values were used in this study to correct the bias. HDL-C, high-density lipoprotein cholesterol; SD, standard deviation.

However, the effect of vitamin D on HDL-C was less conclusive. Exclusion of the Nazarabadi et al. (2022) study from the meta-analysis substantially reduced heterogeneity (I^2^ decreased from 92% to 18%), revealing a statistically significant improvement in HDL-C levels with vitamin D treatment (MD = 1.43, 95% CI [0.69∼2.18], *P* = 0.0002) (Fig. 4). Subgroup analysis further demonstrated that daily vitamin D supplementation significantly increased HDL-C levels in metabolic syndrome patients (MD = 1.77, 95% CI [0.90∼2.64] *P* < 0.0001, I^2^=35%) (Fig. 5A). In contrast, weekly regimens did not exhibit a significant benefit on HDL-C (MD = 2.10, 95% CI [−2.94∼7.14] *P* = 0.41, I^2^ = 95%) (Fig. 5B).

Regardless of whether the regimen was administered daily or weekly, vitamin D supplementation showed no significant benefit in improving LDL-C and TC levels in patients with metabolic syndrome (all *P* values > 0.05).

## Discussion

Vitamin D is well-established for its role in bone metabolism; In recent years, its extraskeletal roles have also garnered increasing attention. The effects of vitamin D on metabolic syndrome and its constituent factors, such as blood glucose, blood pressure, lipids, and body weight, have been inconsistent across studies (Alves et al., 2021; Ebadi et al., 2021; Rajaie et al., 2021; Sardar et al., 2022; Harreiter et al., 2022). Some studies have also revealed a link between vitamin D and autoimmunity, infections, respiratory diseases, and cancer morbidity and mortality (Giustina et al., 2024).

Our meta-analysis demonstrated the beneficial effects of vitamin D on TG control in patients with MetS. The inclusion of the Kelishadi et al. (2014) introduced significant heterogeneity, which might be attributed to its younger participant population. Further RCTs are needed to explore if the benefit of vitamin D on TG levels differs between adults and minors. Notably, the positive effect of vitamin D on TG regulation remained significant regardless of whether the Kelishadi et al. (2014) study was included, demonstrating its consistent benefit across both minors and adults.

However, different kinds of patients may respond differently to vitamin D supplementation. For instance, adolescents with active bone metabolism and postmenopausal women at risk of osteoporosis likely require different vitamin D supplementation strategies. In addition, body weight may be another influential factor, with suggestions that vitamin D dosage for individuals with obesity should be “two to three times” greater than the recommended dose for individuals with normal body weight (Holick et al., 2011).

Therefore, in patients with MetS, especially those with elevated TG and low vitamin D levels, more aggressive vitamin D supplementation is recommended. The dosage of vitamin D supplementation should also take into account factors such as the patient’s age, body weight, and baseline vitamin D levels.

The beneficial effect of vitamin D on HDL-C levels is less definitive. Heterogeneity in the results might be attributed to the Nazarabadi et al. (2022) study, and after its exclusion, vitamin D supplementation showed potential benefits for HDL-C improvement. The inclusion of only postmenopausal women in Nazarabadi et al. (2022) study may explain the heterogeneity. Although there was no consensus on the optimal vitamin D supplementation regimen, the beneficial effect on HDL-C levels appeared more pronounced with daily administration.

Currently, there are few drugs specifically indicated for improving low HDL-C levels. Investigating the effect of vitamin D supplementation on HDL-C improvement is of great significance, thus, the proactive use of vitamin D is recommended in patients with metabolic syndrome who have low vitamin D levels and require HDL-C improvement.

Vitamin D supplementation may influence TG levels through the following mechanisms:

Metabolic syndrome is characterized by insulin resistance (Saklayen, 2018). In the prediabetic state, selective muscle insulin resistance diverts ingested glucose to the liver, stimulating hepatic de novo lipogenesis. This process leads to hypertriglyceridemia and reduced plasma HDL levels. Insulin resistance also increases adipose tissue lipolysis, elevating fatty acid delivery to the liver and promoting hepatic triglyceride synthesis (Samuel & Shulman, 2016). Vitamin D improves insulin sensitivity and suppresses triglyceride production (Szymczak-Pajor, Drzewoski & Śliwińska, 2020).

1,25(OH)_2_D is the primary ligand of the vitamin D receptor (VDR). The widespread distribution of VDR, including in liver, muscle and fat tissue (Kirwan, 2023; Spyksma et al., 2024), may account for vitamin D’s multifaceted effects. The genomic actions of vitamin D mediated by VDR are believed to drive a large share of its biological impacts in health and disease, particularly in the context of obesity (Bennour et al., 2022). Notably, 43% of VDR target genes are involved with metabolism (Saccone, Asani & Bornman, 2015). Additionally, non-genomic effects of vitamin D have also been reported.

Several studies have highlighted two health effects of 1,25(OH)_2_D that are highly relevant in the setting of adiposity: firstly, the relation to inflammation and, secondly, the effect on adipokine secretion, both of which could affect the lipid levels in patients with MetS (Spyksma et al., 2024).

There were several limitations in this meta-analysis.

First, some included studies exhibited imbalanced baseline lipid levels, potentially introducing bias. We utilized change-from-baseline values for meta-analysis to mitigate the bias. And mixing of post-intervention value scores and change-from-baseline values is not a problem when it comes to meta-analysis of mean difference, according to the Cochrane Handbook for Systematic Reviews of Interventions (Higgins et al., 2024). The consistency of results, both with and without studies featuring imbalanced baselines, supported the reliability of our conclusions.

Second, the included studies used different vitamin D supplementation regimens. However, as shown in Tables 1 and 3, all reported baseline mean/median 25(OH)D levels of included participants were below 20 ng/ml, which is commonly defined as vitamin D deficiency (Płudowski et al., 2023). Regardless of the regimen, supplementation increased 25(OH)D levels above 20ng/ml in treatment groups, confirming intervention efficacy and mitigating potential bias. This finding, along with low heterogeneity, strengthened the reliability of the results.

Third, some studies were limited by small sample size. Therefore, future high quality RCTs are needed to strengthen the evidence base.

Fourth, more RCTs need to be included to conduct more in-depth analyses, thereby identifying an optimal vitamin D administration regimen for improving blood lipid profiles. We call for more subsequent studies to be carried out in this field, as this would be of great clinical significance.

## Conclusions

This meta-analysis highlights the potential of vitamin D supplementation as an adjunctive therapeutic strategy for lipid management in patients with metabolic syndrome. Our meta-analysis confirmed the effects of vitamin D in the management of TG levels in metabolic syndrome patients. Vitamin D supplementation showed potential benefits on increasing the HDL-C levels especially with daily regimens. Although no significant effects were observed on TC and LDL-C, the overall positive impact on TG and HDL-C levels suggests that vitamin D supplementation could serve as a valuable ancillary intervention to improve dyslipidemia in patients with MetS. More studies are needed to corroborate these findings and to identify the optimal vitamin D administration regimen.

##  Supplemental Information

10.7717/peerj.21086/supp-1 Supplemental Information 1 PRISMA checklist

10.7717/peerj.21086/supp-2 Supplemental Information 2 Raw data

10.7717/peerj.21086/supp-3 Supplemental Information 3 The research strategy

10.7717/peerj.21086/supp-4 Supplemental Information 4 Funnel plot of mean difference (MD) for the effect of vitamin D therapy on triglyceride (TG)

10.7717/peerj.21086/supp-5 Supplemental Information 5 Meta-analysis of effects of vitamin D on TC level TC: total cholesterol; SD: standard deviation.

10.7717/peerj.21086/supp-6 Supplemental Information 6 Meta-analysis of effects of vitamin D on HDL-C level The baseline HDL-C levels were significantly different between intervention and control groups in study (Farag et al., 2019), thus, the change-from-baseline values were used in this study to correct the bias. HDL-C: high-density lipoprotein cholesterol; SD: standard deviation.

10.7717/peerj.21086/supp-7 Supplemental Information 7 Meta-analysis of effects of vitamin D on LDL-C level LDL-C: low-density lipoprotein cholesterol; SD: standard deviation.

## Additional Information and Declarations

### Data Availability

The following information was supplied regarding data availability: This is a Systematic Review/Meta-analysis.
