# Supplementary material for: Effect of vitamin D supplementation on blood lipids in patients with metabolic syndrome: a meta-analysis
Source: PeerJ. 2026 Apr 13;14:e21086. doi: 10.7717/peerj.21086 (PMC13086024; doi:10.7717/peerj.21086)
Supplement: Supplemental Information 3 [file peerj-14-21086-s003.docx]

The literature search was conducted in two phases: an initial search (up to November 9, 2024) and an update search (November 9, 2024 to February 1, 2026). The search strategy for the update search was identical to that of the initial search, with the only modification being the restriction of the publication date to the specified range (November 9, 2024–February 1, 2026, or 2024–2026).

**PubMed**

#1 "Metabolic Syndrome"[MH] OR "Metabolic Diseases"[MH] OR "Metabolic Syndrome"[TW] OR "metabolic disease"[TW] OR "metabolic disorder"[TW]

#2 "Lipid Metabolism Disorders"[MH] OR "Dyslipidemias"[MH] OR "Hypertriglyceridemia"[MH] OR "lipoproteins, hdl"[MH] OR "Triglycerides"[MH] OR "Hyperlipidemias"[TW] OR "Lipid Metabolism Disorders"[TW] OR "Dyslipidemias"[TW] OR "Hypertriglyceridemia"[TW] OR "Lipoproteins"[TW] OR "HDL"[TW] OR "Triglycerides"[TW] OR "high triglycerides"[TW] OR "low hdl c"[TW] OR "low high density lipoprotein cholesterol"[TW] OR "high density lipoprotein cholesterol"[TW]

#3 "Vitamin D"[MH] OR "Cholecalciferol"[MH] OR "Hydroxycholecalciferols"[MH] OR "Calcifediol"[MH] OR "Dihydroxycholecalciferols"[MH] OR "Calcitriol"[MH] OR "Ergocalciferols"[MH] OR "25-Hydroxyvitamin D 2"[MH] OR "Dihydrotachysterol"[MH]

#4 "Cholecalciferol"[TW] OR "vitamin d*"[TW]

#5 "Hydroxycholecalciferols"[TW] OR "hydroxyvitamins d*"[TW] OR "Alfacalcidol"[TW] OR "1 alpha hydroxyvitamin d*"[TW] OR "1-hydroxycholecalciferol"[TW] OR "1 alpha-hydroxycholecalciferol"[TW] OR "Calcifediol"[TW] OR "25 hydroxyvitamin d*"[TW] OR "Calcidiol"[TW] OR "25-hydroxycholecalciferol"[TW]

#6 "dihydroxycholecalciferol*"[TW] OR "dihydroxyvitamin d*"[TW] OR "Calcitriol"[TW] OR "1 25 oh 2d3"[TW] OR "1,25-Dihydroxycholecalciferol"[TW] OR "1,25-dihydroxyvitamin D3"[TW] OR "1 alpha,25-dihydroxyvitamin D3"[TW] OR "1 alpha,25-dihydroxycholecalciferol"[TW]

#7 "ergocalciferol*"[TW] OR "calciferol*"[TW] OR "25-hydroxyergocalciferol"[TW] OR "25-hydroxycalciferol"[TW] OR "Dihydrotachysterol"[TW]

#8 #3 or #4 or #5 or #6 or #7

#9"randomized controlled trial"[PT]

#10 "controlled clinical trial"[PT]

#11 "randomized"[TIAB]

#12 "placebo"[ TIAB]

#13 "drug therapy"[SH]

#14 "randomly"[TIAB]

#15 "trial"[TIAB]

#16 "groups"[TIAB])

#17 #9 or #10 or #11 or #12 or #13 or #14 or #15 or #16

#18"Animals"[MH] NOT "Humans"[MH]

#19 #17 not #18

#20 #1 and #2 and #8 and #19

**Cochrane(central):**

#1 MeSH descriptor: [Vitamin D] explode all trees

#2 MeSH descriptor: [Cholecalciferol] explode all trees

#3 MeSH descriptor: [Hydroxycholecalciferols] explode all trees

#4 MeSH descriptor: [Calcifediol] explode all trees

#5 MeSH descriptor: [Dihydroxycholecalciferols] explode all trees

#6 MeSH descriptor: [Calcitriol] explode all trees

#7 MeSH descriptor: [Ergocalciferols] explode all trees

#8 MeSH descriptor: [25-Hydroxyvitamin D 2] explode all trees

#9 MeSH descriptor: [Dihydrotachysterol] explode all trees

#10 ("Vitamin D" or "Cholecalciferol" or "Vitamin D3"):ti,ab,kw (Word variations have been searched)

#11 ("Hydroxycholecalciferols" or "Hydroxyvitamins D" or "Alfacalcidol" or "1 alpha-hydroxyvitamin D3" or "1-hydroxycholecalciferol" or "1 alpha-hydroxycholecalciferol" or "Calcifediol" or "25-Hydroxyvitamin D3" or "Calcidiol" or "25-hydroxycholecalciferol"):ti,ab,kw (Word variations have been searched)

#12 ("Dihydroxycholecalciferols" or "Dihydroxyvitamin D" or "Calcitriol" or "1,25-(OH)2D3" or "1,25-Dihydroxycholecalciferol" or "1,25-dihydroxyvitamin D3" or "1 alpha,25-dihydroxyvitamin D3" or "1 alpha,25-dihydroxycholecalciferol"):ti,ab,kw OR ("Ergocalciferols" or "Vitamin D2" or "Calciferols" or "25-Hydroxyvitamin D2" or "25-hydroxyergocalciferol" or "25-hydroxycalciferol" or "Dihydrotachysterol"):ti,ab,kw (Word variations have been searched)

#13 #1 or #2 or #3 or #4 or #5 or #6 or #7 or #8 or #9 or #10 or #11 or #12

#14 MeSH descriptor: [Metabolic Syndrome] explode all trees

#15 MeSH descriptor: [Metabolic Diseases] explode all trees

#16 ("metabolic syndrome" or "metabolic disease" or "metabolic disorder"):ti,ab,kw

#17 #14 or #15 or #16

#18 MeSH descriptor: [Hyperlipidemias] explode all trees

#19 MeSH descriptor: [Lipid Metabolism Disorders] explode all trees

#20 MeSH descriptor: [Dyslipidemias] explode all trees

#21 MeSH descriptor: [Hypertriglyceridemia] explode all trees

#22 MeSH descriptor: [Lipoproteins, HDL] explode all trees

#23 MeSH descriptor: [Triglycerides] explode all trees

#24 ("hyperlipidemias" or "lipid metabolism disorders" or "dyslipidemias" or "hypertriglyceridemia" or "Lipoproteins, HDL" or "Triglycerides" or "high triglycerides" or "low HDL-C" or "low high density lipoprotein cholesterol" or "high density lipoprotein cholesterol"):ti,ab,kw

#25 #18 or #19 or #20 or #21 or #22 or #23 or #24

#26 #13 and #17 and #25

#27 #26 in Trials

**EMBASE:**

#33. #3 AND #6 AND #12 AND #32

#32. #27 NOT #31

#31. #28 NOT #30

#30. #28 AND #29

#29. 'human'/exp

#28. 'animal'/exp OR 'animal experiment'/exp

#27. #13 OR #14 OR #15 OR #16 OR #17 OR #18 OR #19 OR #20 OR #21 OR #22 OR #23 OR #24 OR #25 OR #26

#26. placebo*:ab,ti

#25. 'placebo'/exp

#24. (clin* NEAR/3 trial*):ab,ti

#23. 'clinical trial'/exp

#22. 'latin square design'/exp

#21. 'control group'/exp

#20. 'crossover procedure'/exp

#19. 'experimental design'/exp

#18. ((singl* OR doubl* OR trebl* OR tripl*) NEAR/3 (blind* OR mask*)):ab,ti

#17. 'single blind procedure'/exp

#16. 'double blind procedure'/exp

#15. random*:ab,ti

#14. 'randomization'/exp

#13. 'randomized controlled trial'/exp

#12. #7 OR #8 OR #9 OR #10 OR #11

#11. 'ergocalciferols' OR 'vitamin d2' OR 'calciferols' OR '25-hydroxyvitamin d2' OR '25-hydroxyergocalciferol' OR '25-hydroxycalciferol' OR 'dihydrotachysterol'

#10. 'dihydroxycholecalciferols' OR 'dihydroxyvitamin d' OR 'calcitriol' OR '1,25-(oh)2d3' OR

'1,25-dihydroxycholecalciferol' OR '1,25-dihydroxyvitamin d3' OR '1 alpha,25-dihydroxyvitamin d3' OR '1 alpha,25-dihydroxycholecalciferol'

#9. 'hydroxycholecalciferols' OR 'hydroxyvitamins d' OR 'alfacalcidol' OR '1 alpha-hydroxyvitamin d3' OR '1-hydroxycholecalciferol' OR '1 alpha-hydroxycholecalciferol' OR 'calcifediol' OR '25-hydroxyvitamin d3' OR 'calcidiol' OR '25-hydroxycholecalciferol'

#8. (vitamin NEXT/2 d*) OR 'vitamin d' OR 'cholecalciferol'

#7. 'vitamin d'/exp OR 'cholecalciferol'/exp OR 'hydroxycholecalciferols'/exp OR 'calcifediol'/exp OR 'dihydroxycholecalciferols'/exp OR 'calcitriol'/exp OR 'ergocalciferols'/exp OR '25-hydroxyvitamin d 2'/exp OR 'dihydrotachysterol'/exp

#6. #4 OR #5

#5. 'hyperlipidemias' OR 'lipid metabolism disorders' OR dyslipidemias OR hypertriglyceridemia OR 'lipoproteins, hdl' OR triglycerides OR 'high triglycerides' OR 'low hdl-c' OR 'low high density lipoprotein cholesterol' OR 'high density lipoprotein cholesterol'

#4. 'hyperlipidemias'/exp OR 'lipid metabolism disorders'/exp OR 'dyslipidemias'/exp OR 'hypertriglyceridemia'/exp OR 'lipoproteins, hdl'/exp OR 'triglycerides'/exp

#3. #1 OR #2

#2. 'metabolic syndrome' OR 'metabolic disease' OR 'metabolic disorder'

#1. 'metabolic syndrome'/exp OR 'metabolic diseases'/exp

**SinoMed** (An English translation of the search strategy has been attached)

1. "维生素D"[不加权:扩展] OR "骨化二醇"[不加权:扩展] OR "骨化三醇"[不加权:扩展] OR "维生素D"[常用字段:智能] OR "骨化二醇"[常用字段:智能] OR "骨化三醇"[常用字段:智能] OR "阿法骨化醇"[常用字段:智能] OR "双氢速甾醇"[常用字段:智能] OR "麦角钙化醇"[常用字段:智能]
2. "代谢综合征"[不加权:扩展] OR "代谢综合征"[常用字段:智能]
3. "高脂血症"[不加权:扩展] OR "甘油三酯"[常用字段:智能] OR "胆固醇"[常用字段:智能]
4. (#3) AND (#2) AND (#1)

限定检索：（临床试验 OR随机对照试验 OR多中心研究）AND 人类

**SinoMed** (Translation)

#1. "Vitamin D"[Unweighted: Expanded] OR "Calcifediol"[Unweighted: Expanded] OR "Calcitriol"[Unweighted: Expanded] OR "Vitamin D"[Common Fields: Smart] OR "Calcifediol"[Common Fields: Smart] OR "Calcitriol"[Common Fields: Smart] OR "Alfacalcidol"[Common Fields: Smart] OR "Dihydrotachysterol"[Common Fields: Smart] OR "Ergocalciferol"[Common Fields: Smart]

#2. "Metabolic Syndrome"[Unweighted: Expanded] OR "Metabolic Syndrome"[Common Fields: Smart]

#3. "Hyperlipidemia"[Unweighted: Expanded] OR "Triglycerides"[Common Fields: Smart] OR "Cholesterol"[Common Fields: Smart]

#4. #3 AND #2 AND #1

Filters: (Clinical Trial OR Randomized Controlled Trial OR Multicenter Study) AND Humans
