# Supplementary figures and images for: Effect of vitamin D supplementation on blood lipids in patients with metabolic syndrome: a meta-analysis
Source: PeerJ. 2026 Apr 13;14:e21086. doi: 10.7717/peerj.21086 (PMC13086024; doi:10.7717/peerj.21086)

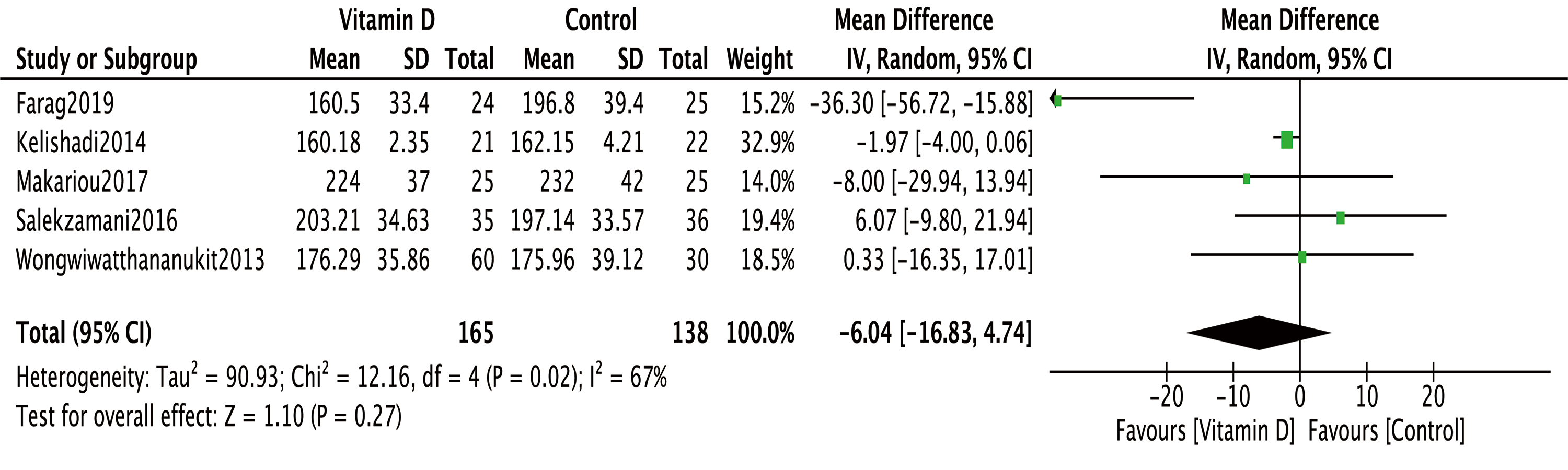

Supplement: Supplemental Information 5 — TC: total cholesterol; SD: standard deviation. [file peerj-14-21086-s005.png]

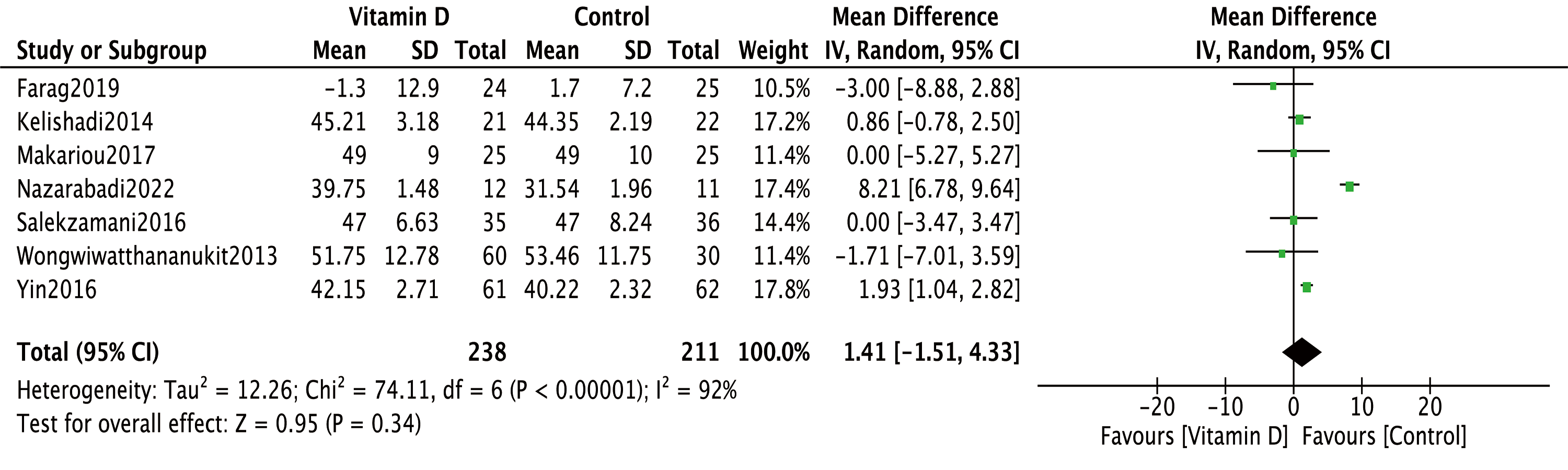

Supplement: Supplemental Information 6 — The baseline HDL-C levels were significantly different between intervention and control groups in study (Farag et al., 2019), thus, the change-from-baseline values were used in this study to correct the bias. HDL-C: high-density lipoprotein cholesterol; SD: standard deviation. [file peerj-14-21086-s006.png]

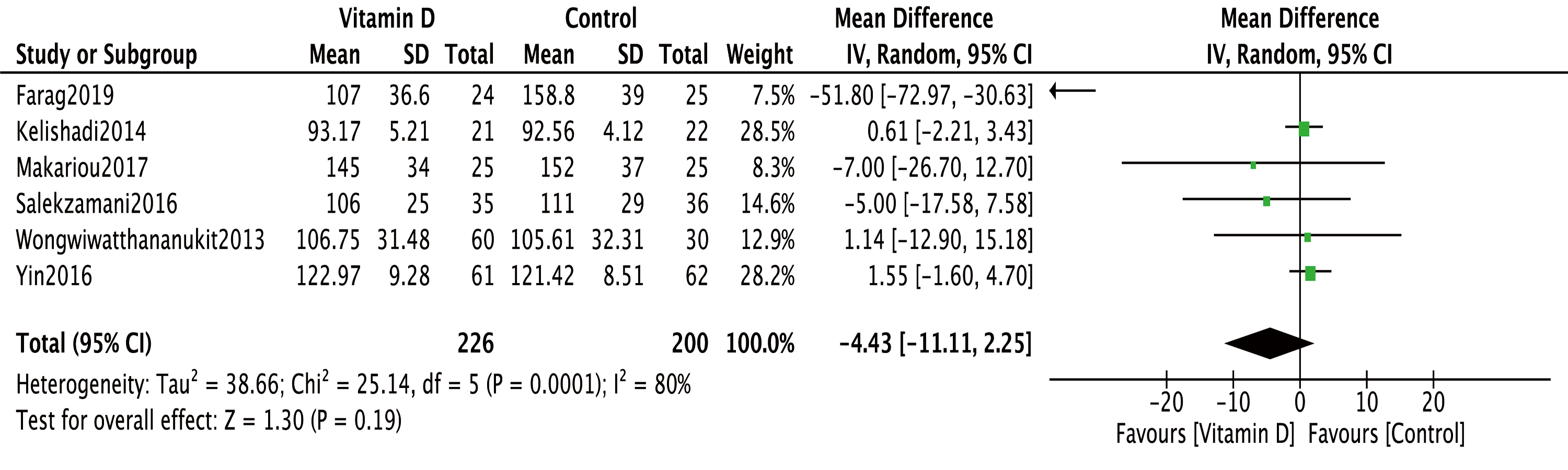

Supplement: Supplemental Information 7 — LDL-C: low-density lipoprotein cholesterol; SD: standard deviation. [file peerj-14-21086-s007.png]
